# A randomized, double‐blind, placebo‐controlled phase 2 study of SHR‐1707, an anti‐amyloid‐β antibody, in patients with mild cognitive impairment and mild Alzheimer's disease

**DOI:** 10.1002/alz70859_100046

**Published:** 2025-12-25

**Authors:** Jiong Shi, Yun Xu, Shenyu Zhao, Jin‐Tai Yu, Kai Wang, Jiehao Zhao, Yi Li, Chunming Xie, Qing'e Zhang, Qidong Chen, Xia Li, Guoping Peng, Li Yang, Yong Ji, Zhaozhao Cheng, Xinyi Lv, Qiong Wang, Xinning Wang, Junpeng Zhuang, Hongyan Qiu, Ye Xu, Sheng Feng, Yuqi Wang, Gang Cheng

**Affiliations:** ^1^ The First Affiliated Hospital of USTC, Hefei, anhui China; ^2^ Nanjing Drum Tower Hospital Clinical College of Nanjing Medical University, Nanjing, Jiangsu China; ^3^ The First Affiliated Hospital of Zhengzhou University, Zhengzhou, Henan China; ^4^ Huashan Hospital, Fudan University, Shanghai China; ^5^ The First Affiliated Hospital of Anhui Medical University, Hefei China; ^6^ Guangdong Provincial People's Hospital, Guangzhou China; ^7^ Qilu Hospital of Shandong University, Jinan China; ^8^ Zhongda Hospital Southeast University, Nanjing China; ^9^ Beijing Anding Hospital Catital Medical University, Beijing China; ^10^ Beijing Tiantan Hospital Catital Medical University, Beijing China; ^11^ Shanghai Mental Health Center, Shanghai Jiao Tong University School of Medicine, Shanghai China; ^12^ The First Affiliated Hospital, Zhejiang University School of Medicine, Hangzhou, Zhejiang China; ^13^ Tianjin Medical University General Hospital, Tianjin China; ^14^ Tianjin Huanhu Hospital, Tianjin China; ^15^ The First Affiliated Hospital of USTC, Hefei China; ^16^ Jiangsu Hengrui Pharmaceuticals Co., Ltd., Shanghai China

## Abstract

**Background:**

SHR‐1707 is a humanized IgG1 monoclonal antibody against amyloid‐β (Aβ). In vitro, SHR‐1707 displayed a similar affinity for Aβ fibrils and protofibrils, and a relatively higher affinity for Aβ monomers than lecanemab. In the 5xFAD transgenic mouse model of Alzheimer's disease (AD), SHR‐1707 safely reduced brain Aβ deposition and ameliorated memory deficits. Two phase 1 studies demonstrated that a single dose of SHR‐1707 at 2–60 mg/kg was safe and well‐tolerated in healthy young adult and elderly subjects (*Alz Res Therapy* 16, 218 (2024)). In a phase 1b trial involving patients with mild cognitive impairment (MCI) due to AD or mild AD, SHR‐1707 at doses of ≤10 mg/kg every 2 weeks (Q2W) for 26 weeks was generally well‐tolerated. Doses of 5–20 mg/kg of SHR‐1707 reduced amyloid in a dose‐dependent manner than placebo. These preclinical and clinical findings provide strong support for further development of SHR‐1707.

**Method:**

This was a randomized, double‐blind, placebo‐controlled phase 2 study (NCT06199037). Patients with MCI due to AD or mild AD aged 50‐85 years would be randomized (2:1) to receive intravenous SHR‐1707 at 10 mg/kg Q2W or placebo for 26 weeks, stratified by ApoE ε4 status (carriers versus non‐carriers). After the double‐blind period, patients who voluntarily continued to participate in the study would enter the open‐label extension period, in which patients would be given SHR‐1707 at 10 mg/kg Q2W for 52 weeks. Primary endpoint was changes from baseline in cerebral Aβ deposition at Week 26 as measured by amyloid positron emission tomography imaging. Secondary endpoints were safety, change from baseline in the Clinical Dementia Rating‐Sum of Box at Week 26, changes from baseline in cerebral Aβ deposition at Weeks 52 and 78, serum SHR‐1707 concentration from first dose to Week 26, and anti‐SHR‐1707 antibody from first dose to Week 26 (incidence and time to onset).

**Result:**

The study is ongoing in China. Topline results are expected in Q2 2025.

**Conclusion:**

The study will investigate the long‐term efficacy and safety of SHR‐1707.

